# Pre-Harvest MeJA Application Counteracts the Deleterious Impact of Al and Mn Toxicity in Highbush Blueberry Grown in Acid Soils

**DOI:** 10.3390/plants10122730

**Published:** 2021-12-11

**Authors:** Jorge González-Villagra, Rocio Pino, Claudio Inostroza-Blancheteau, Paula Cartes, Alejandra Ribera-Fonseca, Marjorie Reyes-Díaz

**Affiliations:** 1 Departamento de Ciencias Agropecuarias y Acuícolas, Facultad de Recursos Naturales, Universidad Católica de Temuco, Temuco P.O. Box 15-D, Chile; jorge.gonzalez@uct.cl (J.G.-V.); claudio.inostroza@uct.cl (C.I.-B.); 2 Núcleo de Investigación en Producción Alimentaria, Facultad de Recursos Naturales, Universidad Católica de Temuco, Temuco P.O. Box 15-D, Chile; 3 Carrera de Agronomía, Facultad de Ciencias Agropecuarias y Forestales, Universidad de La Frontera, Avenida Francisco Salazar 01145, Temuco P.O. Box 54-D, Chile; r.pino03@ufromail.cl; 4 Center of Plant-Soil Interaction and Natural Resources Biotechnology, Scientific and Technological Bioresource Nucleus (BIOREN), Universidad de La Frontera, Temuco P.O. Box 54-D, Chile; paula.cartes@ufrontera.cl; 5 Departamento de Ciencias Químicas y Recursos Naturales, Facultad de Ingeniería y Ciencias, Universidad de La Frontera, Temuco P.O. Box 54-D, Chile; 6 Centro de Fruticultura, Facultad de Ciencias Agropecuarias y Forestales, Universidad de La Frontera, Avenida Francisco Salazar 01145, Temuco P.O. Box 54-D, Chile

**Keywords:** CO_2_ assimilation, fruit quality, plant growth, plant hormone, total phenols

## Abstract

Volcanic ash-derived soils are characterized by low pH (pH ≤ 5.5) with increased concentrations of aluminum (Al^3+^) and manganese (Mn^2+^), which decreases plant growth, fruit quality, and yield. Methyl jasmonate (MeJA) improves abiotic stress tolerance. Our work aimed to evaluate the application of MeJA’s impact on the growth, antioxidant defense, and fruit quality of highbush blueberry grown under Al and Mn toxicity. A field assay was conducted with four-year-old bushes of highbush blueberry cultivar Legacy under eight treatments (Control, Al (87% of Al saturation), Mn (240 mg kg^−1^), and Al–Mn with and without MeJA application). Physiological, biochemical, and fruit quality parameters were measured. Growth rate significantly decreased with Al (20%), Mn (45%), and Al–Mn (40%). MeJA application recovered the growth rate. Photosynthetic parameters were not affected. Antioxidant activity increased under all treatments compared with controls, being higher with MeJA application. Total phenols (TP) were decreased in plants under Al (43%) and Mn (20%) compared with controls. MeJA application increased TP in all treatments. Fruits of bushes under Al and Mn toxicity with MeJA applications exhibited an increase in fruit firmness and weight, maintaining suitable contents of soluble solids. Our results provide insights about the beneficial effect of MeJA application on growth, antioxidant properties, and fruit quality of highbush blueberry plants grown in acid soils under Al and Mn toxicity.

## 1. Introduction

Highbush blueberry (*Vaccinium corymbosum* L.) production is an important agronomic activity in South Central and South of Chile. Nonetheless, in this zone blueberry grows on volcanic ash-derived soils (Andisols) characterized by acidity (pH ≤ 5.5) [1], and by high amounts of aluminum (Al^3+^) and manganese (Mn^2+^), which can provoke negative impacts on the gas exchange, plant growth, fruit quality, and yield of this species [2,3,4]. It has been shown that Al phytotoxicity can induce depolarization of plasma membrane potential and inhibits nutrient uptake and their transport, interfering with membrane-localized solute transporters, such as potassium, calcium, magnesium, nitrate, and ammonium transporters [5,6]. Likewise, Al phytotoxicity increases reactive oxygen species (ROS) production, inducing oxidative stress in blueberry plants, with differential responses among cultivars, and that the resistance levels are strongly related to their antioxidant capacity [3,7]. Thus, Reyes-Díaz et al. [8] showed that the short-term exposure to Al differentially affected photochemical efficiency of PSII of blueberry cultivars, Brigitta being the most resistant cultivar to Al toxicity, closely followed by Legacy, whereas Bluegold was the most sensitive. Much effort and progress has been made in understanding the physiological and molecular mechanisms underlying Al toxicity in plants, e.g., in [9,10]. However, the primary mechanisms remain unclear and speculative [9,10,11,12,13].

In spite of this, Mn is an essential micronutrient that participates actively in several metabolic processes, mainly in photosynthesis; however, when Mn is highly available, it can be toxic for plants [11,12,13] by altering several metabolic processes and increasing ROS, causing oxidative damage and thereby cellular damage [12,14]. Likewise, our studies demonstrated that excess Mn negatively and differently affects the physiological and biochemical features of blueberry cultivars growing under nutrient solution [15,16], with Legacy and Brigitta being identified as Mn-resistant cultivars and Bluegold as an Mn-sensitive cultivar [16]. González-Villagra et al. [4] showed that Legacy, Duke, Camellia, and Cargo are Mn-resistant cultivars, whereas Star is Mn-sensitive cultivar. All these studies have demonstrated that the resistance of blueberry to Mn toxicity is related to antioxidants responses. Noteworthy, very few studies about the joint effect of Al and Mn on plants are available [17,18]. Although high concentrations of Mn and Al can co-occur in acid soils, the resistance mechanisms of plants to Al or Mn do not necessarily coincide [19].

On the other hand, phytohormones, including signaling molecules, such as jasmonic acid (JA), are involved in a wide range of plant responses to several abiotic stresses [20,21,22,23]. Earlier studies showed that both pre- and post-harvest applications of the volatile signal molecule methyl jasmonate (MeJA) induce the synthesis of phenolic compounds with antioxidant properties in berries [24,25,26,27]. Previously, we showed that low doses of exogenous MeJA applied simultaneously with toxic Al improve the antioxidant performance of blueberry plants under greenhouse conditions [28]. In agreement, we found that MeJA applications at pre-harvest improved the antioxidant properties of leaf extracts (mainly anthocyanins) of blueberry, increasing their inhibition levels on cancer cell viability and migration [29]. However, less is known about the influence of MeJA on Mn toxicity. Studies related to Al–Mn interaction and their relationship with phytohormones for improving its resistance mechanisms, productivity, and fruit quality of blueberry growing under acid soil conditions are not available yet. The present work aims to study the impact of MeJA application on the growth, antioxidant properties, and fruit quality of highbush blueberry plants grown in acid soils under Al and Mn toxicity conditions.

## 2. Materials and Methods

### 2.1. Plant Growth Conditions and Soil Treatments

The pot field assay was conducted in Maquehue Experimental Station of La Frontera University, located at La Araucanía Region, Chile (38°47′ S, 73°42′ W). Bushes of 4-year-old highbush blueberry plants (cultivar Legacy) were planted in plastic pots containing 30 kg of Andisol soil (Freire Series, with pH values ranging from 5.5 to 5.8) collected in the same Experimental Station. Plants were maintained with a drip irrigation system with 2 L h^−1^ (self-compensating drippers) applied from the end of November to harvest as needed. Agronomical practices (pruning, fertilization, irrigation, and phytosanitary) were performed according to the commercial recommendations.

Four soil treatments were applied: (i) Control—soil without toxic Al and without toxic Mn concentrations; (ii) Al—soil with phytotoxic Al concentrations (by applying Al_2_ (SO_4_)_3_; Al sulfate); (iii) Mn—soil with phytotoxic levels of Mn (by applying Mn sulfate (MnSO_4_)); (iv) Al–Mn—soil with phytotoxic concentrations of Al and Mn, following the methodology reported by Valle et al. [30].

Then, 20 days after the addition of Al and Mn sulfates, soil samples were analyzed again. The data revealed that the application of Al and/or Mn sulfate increased the concentration of exchangeable Al and Mn in soil, respectively, at expected levels. Soil treated only with Al sulfate reached 87% of Al saturation and 37 mg kg^−1^ of Mn^2+^, soil treated only with Mn sulfate reach 240 mg kg^−1^ of Mn^2+^ and 0.12% of Al saturation and soil treated with both Al and Mn sulfates reached 87% of Al saturation and 220 mg kg^−1^ of Mn^2+^. The control soil (without Al or Mn sulfate applications) had low values of Al saturation (0.12%) and Mn^2+^ (37 mg kg^−1^), suitable values of pH (~6.0) and bases sum (11.5 cmol kg^−1^ soil), and 15% of organic matter, these being the optimal soil conditions for growing blueberries. After planting, pots were randomly located and subjected to an acclimatization period until the MeJA application.

### 2.2. Pre-Harvest MeJA Treatments of Highbush Blueberry Plants

A total of 10 plants of each soil treatment described above were sprayed to the canopy with MeJA solution, whereas the other 10 bushes were un-treated with the phytohormone. Briefly, an aqueous solution (distilled water supplemented with Tween 80 at 0.05% *v*/*v*) at a final concentration of 0.01 mM of MeJA was sprayed to the above-ground part of the blueberry bushes, in a single application (moisturizing area of around 150 mL per bush), at the phenological stage of the fruit set. Distilled water plus Tween 80 (0.05% *v*/*v*) was sprayed to MeJA un-treated plants. For this, a 20 L of back-held spray pump was used. The MeJA dose was selected in accordance with previous studies performed by the research group [28,31]. All foliar sprays were performed the same day, early in the morning, using a back-held spray pump. Once the MeJA application was made, plants were rearranged completely at random.

### 2.3. Plant Growth Determination

Every year, the blueberry plants are pruned; we measured the height of one shoot (1-year-old) per bush in order to determine the growth rate during the growing season. It was calculated by relative growth rate (RGR) from the mean natural logarithm-transformed shoot length: RGR = (lnW2) − (lnW1)/(t2 − t1) [32]. For this, the shoot growth of 10 plants of each treatment were measured periodically from 15 days after the MeJA solution was applied to the harvest. Only the first and last measurements were considered for the formula, where the difference of these was divided by the number of days elapsed.

### 2.4. Aluminum and Manganese Determinations in Leaves

Dry samples of leaves were burned to ashes for 8 h at 500 °C, and later digested with 2 M HCl [33]. Aluminum and Mn concentrations were determined using a simultaneous multi-element atomic absorption spectrophotometer (Model 969 atomic absorption spectrometer, Unicam, Cambridge, UK).

### 2.5. Measurement of Phytohormones Plant Concentration

The methodology of solvent-induced phase transition extraction (SIPTE) was used on fresh leaves, as described by Sarwar and Kremer [34]. The samples were concentrated using a Savant SPD11V integrated vacuum concentrator system and resuspended in 1 mL of 30% *v*/*v* MeOH and were measured spectrophotometrically with Salkowski reagent [35].

### 2.6. Photosynthetic Parameters

Three leaves per plant, of ten plants per treatment, were evaluated (the same plants used for growth determinations). CO_2_ assimilation (A), stomatal conductance (g_s_), and transpiration (E) were determined using a portable infrared CO_2_ analyzer equipped with a Licor LR6400 cuvette measurement with its own light source (0–2000 µmol photons m^−2^s^−1^) and its own control of temperature, humidity, and CO_2_ [6].

Chlorophyll concentration was determined on three leaves per plant (10 plants per treatment) by using a Portable Chlorophyll Meter SPAD. In each measurement, 4 SPAD determinations were performed for each leaf; thus, the average of chlorophyll per plant was obtained from 12 different measurements. The data obtained were expressed in the chlorophyll content index (CCI).

Chlorophyll *a* (Chl*a*), chlorophyll b (Chl*b*), and carotenoids were also determined in phase reversed, solvent gradient, and high-performance liquid chromatography (HPLC, Agilent Technologies Inc., San Jose, CA, USA), following the protocol described by García-Plazaola and Becerril [36].

### 2.7. Determination of Antioxidant-Related Parameters

The biochemical parameters related to antioxidant responses of blueberry plants were measured spectrophotometrically (UV/VIS spectrophotometer, model 2800 UV/VIS, UNICO, Fairfield, NJ, USA) in samples of 10 plants per treatment; leaves were collected from the middle of the shoot (1-year-old shoot per plant), 20 days after MeJA application. Once cut, leaves were stored in hermetic bags, maintained at 4 °C and transported to the laboratory. Then, samples were stored at −80 °C to determine total phenols and antioxidant activity, whereas leaves used for lipid peroxidation analysis were stored at −20 °C.

The antioxidant activity of leaves was measured by the radical scavenging activity (RSA) assay, using the free radical 2.2-diphenyl-1-picrylhydrazyl (DPPH), based on the method described by Chinnici et al. [37]. For this, leaf samples were ground in liquid nitrogen and macerated with 1 mL of ethanol (80% *v*/*v* EtOH/water), and then centrifuged. The supernatant was collected, and the absorbance was measured at 515 nm. TROLOX (6-hydroxy-2,5,7,8-tetramethylchroman-2-carboxylic acid) was used as the standard antioxidant molecule.

Total phenols were determined by using the Folin–Ciocalteu reagent, according to the method of Slinkard and Singleton [38]. For the total phenol determination, 0.1 g of leaf sample was macerated with ethanol (80% *v*/*v* EtOH/water), and centrifuged at 10,000× *g* at 4 °C for 5 min. The absorbance of all samples was measured at 765 nm using a UV–VIS spectrophotometer (model 2800 UV/VIS, UNICO, Fairfield, NJ, USA), and the results were expressed as milligrams of chlorogenic acid equivalent per gram of fresh weight (mg CAE/g FW).

As an oxidative stress indicator, the lipid peroxidation of membranes was determined. Briefly, this parameter was determined in extracts of leaves by using thiobarbituric acid reacting substances (TBARS) assay, according to the modified method of Du and Bramlage [39]. Thus, the absorbance was measured at 532,600 nm and 440 nm in a spectrophotometer.

### 2.8. Aluminum and Manganese Concentrations and Quality of Fruits

Fruit parameters were determined in 10 bushes per treatment, by harvest (January) all berries of each plant. The same day of harvest, the collected fruits were stored in 2 kg clamshell (1 per plant) and transported (4 °C) to the Physiology and Fruit Quality of Fruit Crops Laboratory (Maquehue Experimental Station, La Frontera University), in order to determine the fruit parameters. Aluminum and manganese concentration in fruits were determined by burned to ashes for 8 h at 500 °C and later dissolving the ash in HNO_3_ (1:20 *w*/*v*) following the protocol of Karla [40]. Aluminum and Mn concentrations were determined using a simultaneous multi-element atomic absorption spectrophotometer (Model 969 atomic absorption spectrometer, Unicam, Cambridge, UK). Caliber, firmness, and soluble solids content were determined in 10 fruit samples per plant. Briefly, fruit firmness and caliber were measured by using the FirmPro Texturometer (Happyvolt, Santiago, Chile (https://happyvolt.com/firmpro, accessed on 10 November 2021). Finally, soluble solid content was determined through a Portable Refractometer (Digital Thermo-Compensated Refractometer, Hannah Instruments HI96811, Woonsocket, RI, USA), and the results were expressed in degrees Brix (°Brix).

### 2.9. Experimental Design and Statistical Analyses

The experimental design was completely randomized with four soil treatments (control, Al, Mn, and Al–Mn) x two MeJA doses (0 mM and 0.01 mM). All data were subjected to the normality and equal variance with Kolmogorov–Smirnov test before statistical analysis. Then, data were analyzed by two-way ANOVA with the Software SigmaStat version 2.0 (Sigma Stat, San Jose, CA, USA). The differences among the means were analyzed by the Tukey test (*p* ≤ 0.05).

## 3. Results

### 3.1. Growth Rate, Aluminum and Manganese Concentrations of Leaves

**Figure 1:**
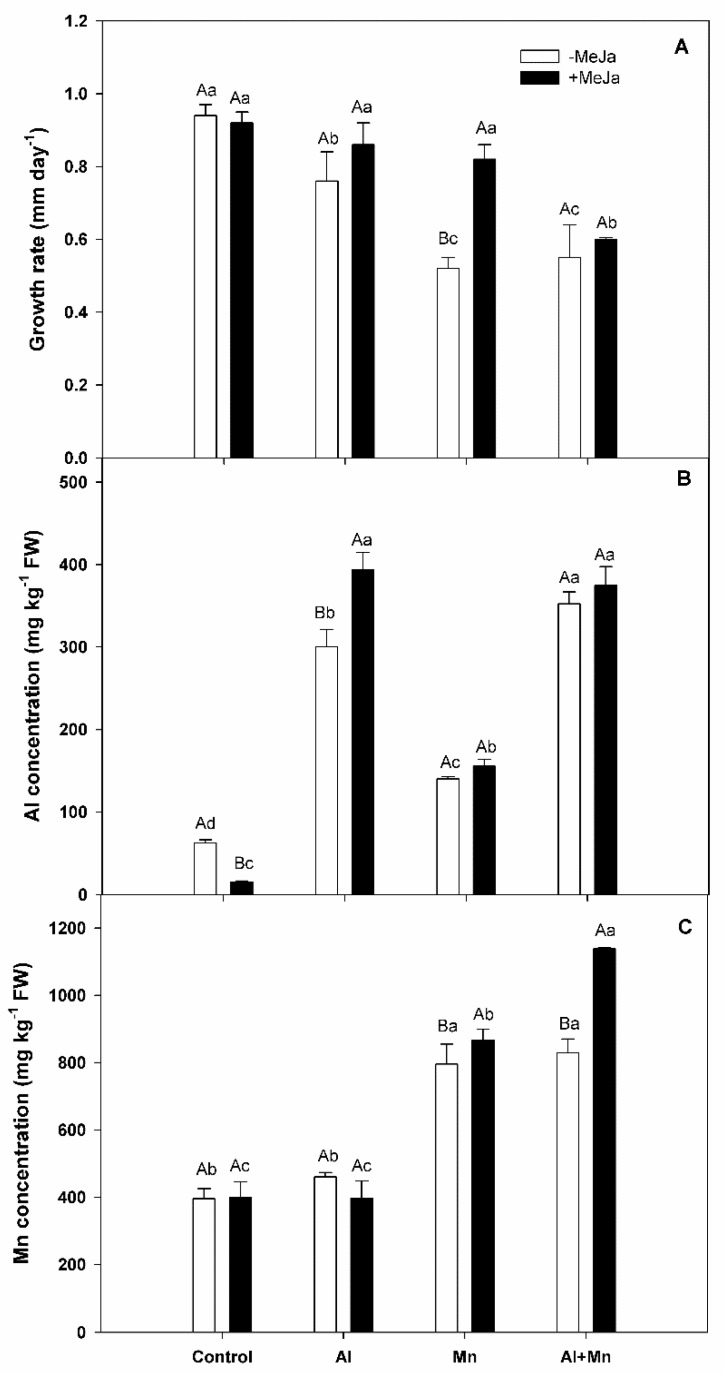
(**A**) Growth rate, (**B**) Al concentration, and (**C**) Mn concentration in leaves of highbush blueberry cv. Legacy plants subjected to different soil treatments (Al, Mn, and Al–Mn toxicity), and exogenous MeJA application. All values represent averages of ten biological replicates ± SE. Different upper-case letters indicate significant differences between MeJA treatments for the same soil conditions according to Tukey’s test (*p* ≤ 0.01). Different lower-case letters indicate significant differences among soil conditions for the same MeJA treatment according to Tukey’s test (*p* ≤ 0.01).

In our study, we observed a significant interaction between soil treatments and MeJA treatments for shoot growth rate, Al leaves concentration, and Mn leaves concentration (*p* ≤ 0.001). Our results revealed that plants grown in soil with either Al, Mn, or Al–Mn significantly decreased growth rate (about 20%, 45%, and 40%, respectively) compared with control plants (Figure 1A). Plants subjected to Mn treatment recovered the growth rate with MeJA application, where a significantly higher (about 40%) growth rate compared with plants without MeJA application was observed. In addition, our results showed that plants subjected to Al or Al–Mn treatments exhibited the highest Al concentration in leaves, which were around 6-fold than that of control plants, followed by Mn treatment (around 3-fold than that of control plants) (Figure 1B). Exogenous MeJA significantly increased by about 25% Al concentration in leaves of plants subjected to Al treatments. By contrast, MeJA decreased (about 75%) Al concentration in leaves of control plants. Meanwhile, plants subjected to Mn or Al–Mn exhibited unchanged Al levels in leaves with MeJA application. As for Mn concentration, plants subjected to Mn or Al–Mn treatments accumulated greater Mn levels (around 2-fold) in leaves compared with control plants (Figure 1C). Meanwhile, plants subjected to Al treatment showed similar Mn levels in leaves as control plants. We observed that plants grown under Mn or Al–Mn subjected to MeJA application increased about 9% and 27% Mn concentration in leaves compared with plants without MeJA application under Mn or Al–Mn treatments, respectively.

### 3.2. Photosynthetic Parameters

**Figure 2:**
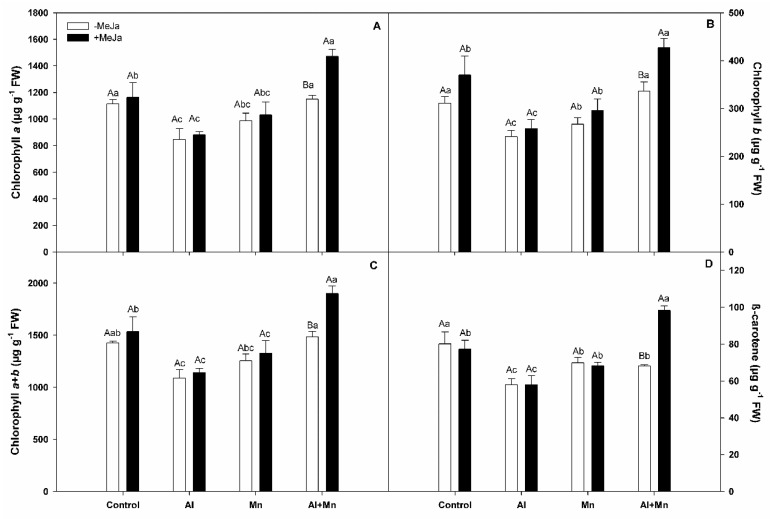
Chlorophylls in highbush blueberry cv. Legacy plants subjected to different soil treatments (Al, Mn, and Al–Mn toxicity), and exogenous MeJA. Chlorophylls *a* (**A**), *b* (**B**), *a* + *b* (**C**), and β-carotene (**D**). All values represent averages of ten biological replicates ± SE. Different upper-case letters indicate significant differences between MeJA treatments for the same soil conditions according to Tukey’s test (*p* ≤ 0.01). Different lower-case letters indicate significant differences among soil conditions for the same MeJA treatment, according to Tukey’s test (*p* ≤ 0.01).

In our study, CO_2_ assimilation showed no significant differences among soil treatments and MeJA application (Table 1). Likewise, g_s_ and *E* had similar behavior, showing no significant differences among soil treatments and MeJA applications (Table 1). We observed that chlorophylls *a*, *b*, and *a* + *b* were significantly decreased (around 20%) in plants subjected to Al and Mn toxicity compared with control plants, without changes with MeJA application (Figure 2). Meanwhile, MeJA-treated plants under the Al–Mn treatment significantly increased chlorophylls *a*, *b*, and *a* + *b*. In addition, our results showed that β-carotene contents decreased in plants subjected to Al, Mn, or Al–Mn soil treatment compared with the control plants, mainly by the effect of Al treatment (Figure 2D). MeJA application only increased carotene levels in plants grown under Al–Mn soil treatment.

### 3.3. Total Phytohormone

**Figure 3:**
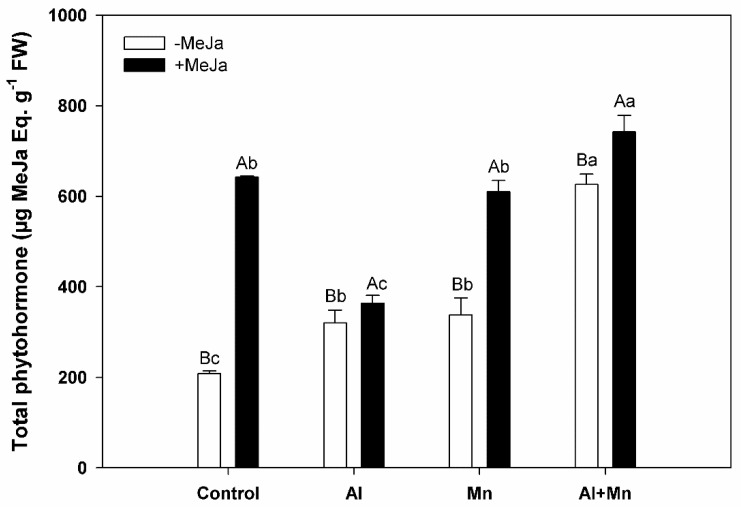
Total phytohormone in leaves of highbush blueberry cv. Legacy plants subjected to different soil treatments (Al, Mn, and Al–Mn toxicity), and exogenous MeJA. All values represent averages of 10 biological replicates ± SE. Different upper-case letters indicate significant differences between MeJA treatments for the same soil conditions according to Tukey’s test (*p* ≤ 0.01). Different lower-case letters indicate significant differences among soil conditions for the same MeJA treatment according to Tukey’s test (*p* ≤ 0.01).

Our results showed a significant interaction between soil treatments and MeJA treatments for total phytohormone. We observed that plants subjected to Al, Mn, or Al–Mn soil treatments significantly increased their total phytohormone levels (35%, 38%, and 300%, respectively) compared with control plants (Figure 3). When MeJA was applied, total phytohormone was increased in plants under all soil treatments. Plants under Al–Mn soil treatment MeJA-treated had the highest total phytohormone level, reaching 742 µg MeJA eq. g^−1^ FW. Meanwhile, the change in total phytohormone was most evident in plants under control treatment with MeJA application, increasing by about 3.1-fold compared with plants under control treatment without MeJA application (Figure 3).

### 3.4. Antioxidant Activity, Total Phenols, and Lipid Peroxidation in Leaves

**Figure 4:**
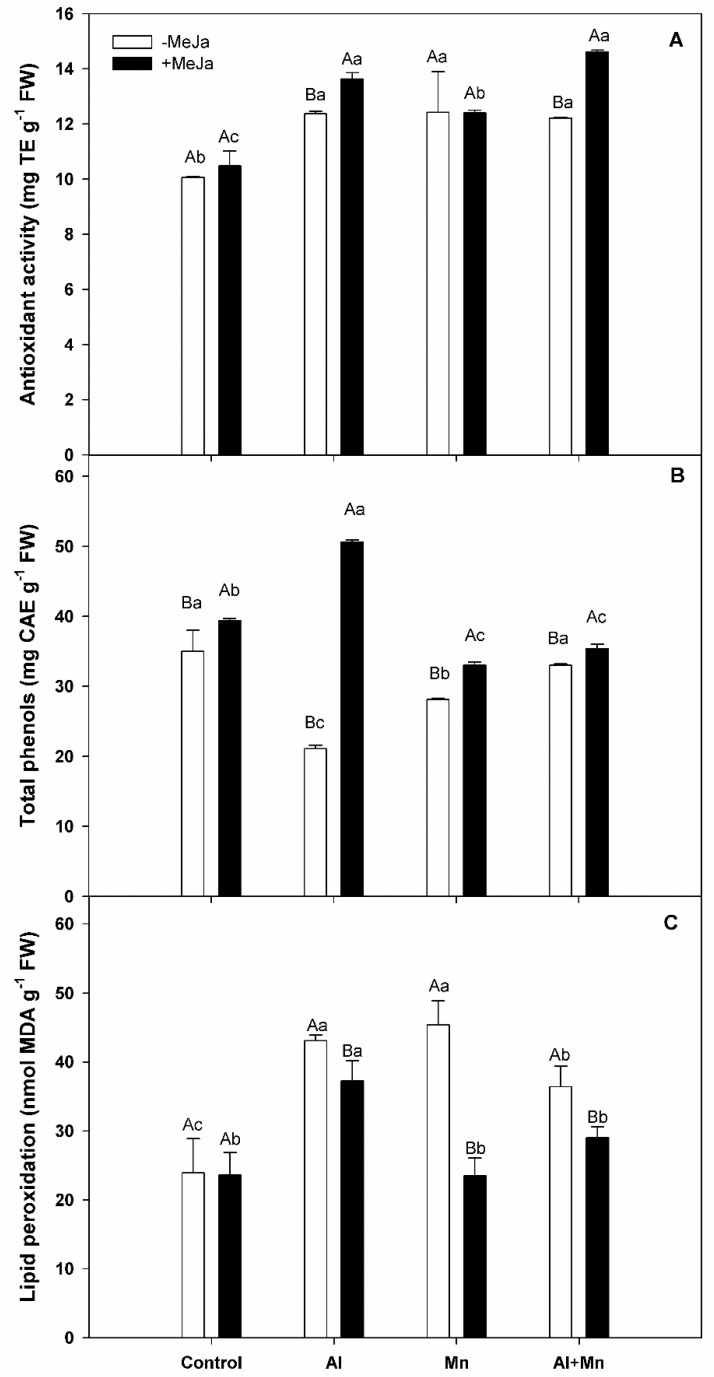
Antioxidant activity (**A**) and total phenols (**B**), and lipid peroxidation (**C**) in leaves of highbush blueberry cv. Legacy plants subjected to different soil treatments (Al, Mn, and Al–Mn toxicity), and exogenous MeJA. All values represent averages of ten biological replicates ± SE. Different upper-case letters indicate significant differences between MeJA treatments for the same soil conditions according to Tukey’s test (*p* ≤ 0.01). Different lower-case letters indicate significant differences among soil conditions for the same MeJA treatment according to Tukey’s test (*p* ≤ 0.01).

In order to evaluate the antioxidant defense system, we determined antioxidant activity (AA), total phenols (TP), and lipid peroxidation in *V. corymbosum* plants (Figure 4). A significant interaction between soil treatments and MeJA treatments for antioxidant activity was observed. Our results revealed that AA levels were about 20% greater in plants under Al, Mn, or Al–Mn soil treatments with respect to control plants (Figure 4A). When MeJA was applied, AA levels were increased (about 10% and 14%) in plants under Al or Al–Mn, with respect to plants under Al or Al–Mn without MeJA application. By contrast, plants under control or Mn soil treatments did not change their AA levels when MeJA was applied. We observed statistically significant interactions between soil treatments and MeJA treatments for total phenols. In our study, total phenols were decreased by about 43% and 20% in plants subjected to Al and Mn soil treatments, respectively, compared with control plants (Figure 4B). When MeJA was applied, all treatments significantly increased their total phenols with respect to their treatments without MeJA application. MeJA-treated plants under Al treatment showed the highest total phenol level and increased 2.5-fold compared with plants subjected to Al without MeJA application.

On the other hand, a significant interaction between soil treatments and MeJA treatments for lipid peroxidation was observed. Lipid peroxidation (LP) increased in response to all treatments without MeJA application, mainly under Mn and Al toxicity, followed by bushes grown under the combination of Al and Mn toxicity (Figure 4C). Furthermore, MeJA application reduced LP levels in all soil treatments with exception of control plants (Figure 4C).

### 3.5. Aluminum and Manganese Concentrations and Quality of Fruits

The aluminum concentration in fruits was increased 2.7-fold and almost 4-fold under Al and Al–Mn treatments, respectively, compared with control. The application of MeJA increased Al concentration by about 1.7-fold and 4-fold in control and Al treatments, respectively (Table 2). It is highlighted that MeJA application decreased (35.6%) Al concentration under Al–Mn treatment. Likewise, Mn concentration was increased under Mn (44.7%) and Al–Mn (3.5-fold) treatments, while MeJA application increased Mn concentration in all treatments compared with control, with the exception of Mn treatment, where the value was maintained (Table 2). The fruit caliber was very similar among soil treatments; it was slightly reduced by the effect of treatments without MeJA application compared with control plants. MeJA supply reduced fruit caliber (11.8%) but only in control plants (Table 2). Total soluble solids (TSS) decreased about 13% under Al–Mn treatment without MeJA application compared with control. Interestingly, MeJA significantly increased TSS in bushes grown under combined Al and Mn toxicity, reaching control values. In the same way, increased berry firmness (23%) was detected in plants grown under combined Al and Mn toxicity with and without MeJA treatment than control plants, while for the other soil treatments, the firmness of fruits was similar, and it did not differ to the control (Table 2).

## 4. Discussion

Aluminum and Mn toxicities are two main constraints to crop production in acid soils [12,41], which can depolarize the plasma membrane and interfere with membrane-localized ion transporters, inhibiting nutrient uptake, photosynthesis, plant growth, and crop yield in higher plants [4,11,12,42,43]. In our study, we found that plants of highbush blueberry cv. Legacy cultivated in soil with phytotoxic concentrations of either Al, Mn, or Al–Mn significantly decreased shoot growth compared with control plants. The greater shoot growth decreases were detected in plants grown under Mn toxicity alone as well as under Al–Mn toxicity, which were 20% higher than those observed for plants subjected to Al excess. Previous studies have shown that the high concentration of Al or Mn in soils causes considerable reductions in photosynthesis and plant growth of highbush blueberries [15,16,44]. However, to date, it is not clear if Mn and Al toxicities are antagonistic or synergistic and if plants with Al-resistance can also resist Mn toxicity. Nonetheless, studies about the combined effect of Al and Mn toxicities in plants mostly reveal that Al toxicity can overcome Mn toxicity in some plant species [17,18,45,46]. In this regard, Blair and Taylor [17] studied the interaction between Al and Mn on growth and metal accumulation in *Triticum aestivum* plants, showing that combined effects of Al and Mn could be described by a multiplicative model; in contrast, metal content in shoots and roots indicated a significant antagonistic effect of Al on the Mn accumulation. In the same context, Yang et al. [18] reported that high levels of Al significantly alleviated Mn toxicity symptoms (including shoot growth inhibition) in *G. max* plants grown under hydroponic conditions, which was related to decreases in Mn concentration in shoots exposed to combined Al and Mn stress. Likewise, Muhammad et al. [46] found that *Hordeum vulgare* plants, grown under combined Al and Mn toxicities, exhibited lower growth reductions, tissue Al and Mn, and photosynthesis than plants grown under Al or Mn treatments alone, demonstrating a clear antagonistic interaction among both metals. Our study found that the shoot growth reductions provoked by the combined action of Al and Mn toxicities on blueberry plants were comparable to those observed in plants grown under Mn toxicity alone, these being 2-fold higher than Al toxicity alone. These findings suggest that: (i) excessive Al availability does not seem to alleviate the Mn toxicity symptoms in blueberry plants, or vice versa, and (ii) the growth reductions observed in response to the combination of Al and Mn toxicity appear to be explained mainly by the effect of Mn excess.

As we mentioned above jasmonic acid (JA) and its derivative methyl jasmonate (MeJA), are phytohormones involved in the regulation of plant responses to biotic and abiotic stresses [47], including heavy metals and metalloids detoxification (reviewed in [48]). Our results showed that MeJA application was capable of recovering growth rate in blueberry bushes subjected to Mn toxicity but not in plants grown under either Al toxicity or Al–Mn stress. On the other hand, plants subjected to either Al toxicity or Al–Mn exhibited the highest leaf Al concentrations, while MeJA supply increased leaf Al of plants subjected to Al toxicity, but it decreased leaf Al of control plants. Our previous findings showed that the pre-harvest application of MeJA to blueberry plants at low doses (5–10 µM) enhances resistance to Al toxicity by marked decreases in lipid peroxidation and Al accumulation, as well as it increases photosynthetic performance compared with non-MeJA-treated plants [28,31]. On the contrary to Al toxicity and phytohormones studies, the influence of MeJA on Mn toxicity is unknown. We found that plants grown under Mn toxicity alone or in combination with Al accumulated higher Mn in comparison with control plants, meanwhile, plants treated with MeJA grown under either Mn or Al–Mn increased leaf Mn concentrations compared with non-MeJA-treated ones. On the other hand, we observed that total phytohormones significantly increased in blueberry plants exposed to either Al, Mn, or the combination of Al and Mn stress, being higher in plants subjected to Al–Mn treatment (until 60% compared with control plants), with values 20% greater than those observed in plants grown under Al or Mn toxicity alone. As expected, when MeJA was applied, total phytohormones increased in plants, independent of the soil treatments applied. Recently, studies performed in *Acutodesmus obliquus*, with applied auxin and cytokinins phytohormones, showed an increase in the phytohormones, regulating the homeostasis of this species, this being an adaptation strategy to heavy metal stress [49]. Thus, although plants exposed to Mn toxicity with MeJA application accumulated higher leaf Mn levels, their higher growth rate could be associated with higher total phytohormone found in highbush blueberry plants.

On the other hand, CO_2_ assimilation and g_s_ were unaffected by any soil treatments or by MeJA application. Nonetheless, chlorophylls *a* and *b* (and Chl *a* + *b*) were decreased in plants grown under Al or Mn toxicity alone compared with control plants, but not changed in response to the combined Al and Mn toxicities. In addition, MeJA application increased chlorophyll contents compared with MeJA untreated plants, but only in bushes grown under Al–Mn toxicity. Moreover, carotene contents decreased in plants grown under all soil toxicity treatments compared with the control plants, mainly by the effect of Al excess. Similarly, to those observed for chlorophylls, MeJA supply significantly increased carotene contents, but only in plants grown under Al–Mn toxicity. In this regard, the beneficial effect of MeJA applications on plants subjected to biotic or abiotic stress, including Al toxicity, has been attributed to the activation of both enzymatic and non-enzymatic antioxidant responses, thus preventing oxidative stress and damages on plasma membranes and photosynthetic apparatus [31,50,51,52]. Pre-harvest MeJA treatments on *Pharbitis nil* produced effects similar to abscisic acid applications, reducing the growth of leaves, roots, buds, and shoots [53], whereas MeJA applications on *G. max* and *Hordeum vulgare* affected the transpiration rate due to stomatal closure [50].

On the other hand, lipid peroxidation of membranes (LPO), measured as an indicator of oxidative stress degree in blueberry leaves, increased in all treatments without MeJA application compared with control plants, mainly in Al and Mn toxicity alone. Thus, we observed that LPO, chlorophylls, and carotene contents were more affected in the treatments with Al or Mn alone compared with the combined Al–Mn treatment. Similar behavior was reported by Yang et al. [18], where Al and Mn toxicity alone showed higher toxicity symptoms compared with the combined Al–Mn toxicity treatment in *G. max* plants. Thus, our results suggest that Al and Mn alone are more toxic than the combined Al–Mn treatment for blueberry plants, showing an antagonistic effect between Al and Mn toxicity. In this sense, Wang et al. [45] suggested that this effect could be attributed to decreased shoot Mn accumulation resulting from an Al-induced decrease in root symplastic Mn uptake, due to an Al-induced change in cell membrane potential. The authors also conclude that Al increased Mn plaques in the roots, and changed the binding properties of the cell wall, resulting in accumulation of non-available Mn in roots. Furthermore, it is noteworthy that the decrease in LPO levels in all treatments, due to the MeJA application, was concomitant with the induction of antioxidant systems (phenols). This agrees with the reported by Ulloa-Inostroza et al. [31], where the application of MeJA in *V. corymbosum* plants subjected to Al toxicity significantly increased antioxidant activity and total phenols, reducing oxidative damage. Previous studies have shown that MeJA application enhanced antioxidant activity and total phenols contents in several species such as *Brassica napus, Brassica juncea,* and *Vigna radiata* [54,55,56]. In this way, preharvest application of MeJA in *Rubus idaeus* plants resulted in a significant increase in phenol compounds such as ellagic acid, quercetin, and myricetin, which was explained by a promoting effect of MeJA on phenyl ammonia lyase (PAL) enzyme activity [57]. Therefore, the reduced LPO levels under Al, Mn, and Al–Mn treatments could be associated with the higher total phenols and greater total phytohormone levels observed in our study.

Most studies suggest that pre-harvest MeJA applications could be a promising tool for increasing fruit quality, but the optimum concentration of this hormone is species- and cultivar-dependent. In the present work, results revealed that total soluble solids (TSS) did not change among the soil treatments. Bushes grown under Al–Mn toxicity without MeJA application decreased TSS compared with control, while MeJA application recovered TSS, reaching control values. In contrast, slight reductions in TSS were observed in response to the phytohormone for the rest of the treatments. Despite fruit caliber being very similar among the soil treatments, this was slightly reduced by the effect of Mn toxicity compared with control plants. Interestingly, the higher values of berry firmness (until 30.5%) were detected for plants grown under Al–Mn toxicity with and without MeJA application, while for the rest of soil treatments, including the control, firmness of fruits were comparable. Kucuker et al. [58] reported that MeJA-treated trees of *Prunus salicina* had higher yields and maintained significantly higher flesh firmness than controls; however, the diameters of MeJA-treated fruits were lower than the control fruits. Despite that, the authors indicated that preharvest MeJA treatment during the ripening of plums might be considered as an efficient tool for preserving fruit flesh firmness at commercial harvest. Similar results in the same species were obtained by Martínez-Esplá et al. [59]. It was reported that preharvest application of MeJA also improved the fruit quality of *P. salicina* during postharvest storage, since both non-enzymatic and enzymatic activity were higher in treated than control *P. salicina* during storage, which could account for the delay in the postharvest ripening process and the extension of shelf-life [60]. On the other hand, our results about the Mn concentration of fruits indicated that MeJA slightly increased its concentration. Contrarily, Al concentration in fruits was increased with MeJA application in all soil treatments (up to 4-fold, reaching 47.8 mg kg^−1^ DW) compared with control, except Al–Mn treatment, where MeJA application decreased the Al concentration (35.6%). It is reported that in blueberry fruits, Al concentrations range from 40 to 2900 µg g^−1^. The authors indicated that the toxic Al content in the berry sample was safe for human consumption [61]. Likewise, Zeiner and Cindrić [62] reported 1248 mg/kg of Al in blueberry fruits, indicating that with this value, an 80 kg person should not consume more than 600 g of blueberries per week, until 2 kg, to reach the maximum permitted, considering no other Al sources. Although our results could be considered lower than those reported for blueberry fruits, it is known that several reports relate Al levels to Alzheimer’s disease [63]. Finally, the joint FAO/WHO Expert Committee on Food Additives reduces the provisional tolerable weekly intake value for Al from 7 mg kg^−1^ body weight/week to 1 mg kg^−1^ body weight/week, which should be considered for food consumption [64]. In the same way, it is important to take into account that Al is absorbed in a proportion of 0.1–0.3% by the gastrointestinal tract, which occurs due to lower pH levels in the upper intestine [65].

## 5. Conclusions

This study provides insights into the impact of MeJA exogenous applications on growth, physiological properties, antioxidant properties, and fruit quality of highbush blueberry plants grown in acid soils under Al and Mn toxicity, alone or in combination. Our results showed the following: (i) that the negative impacts of combined Al and Mn toxicity in blueberry plants could be explained by the antagonistic effect, but mainly by the deleterious effects of Mn excess; (ii) that the improvement of non-enzymatic antioxidant performance induced by MeJA supply was able to overcome oxidative damage induced by metal toxicities; (iii) although the MeJA application increases the Al levels in fruits, the values were lower than those reported for blueberry fruits; therefore, its application was useful for improving berry quality in plants exposed to either Al or Mn excess and mostly for their combination.

## Figures and Tables

**Table 1:** Photosynthetic performance in highbush blueberry cv. Legacy plants subjected to different soil treatments (Al, Mn, and Al–Mn toxicity), and exogenous MeJA. All values represent averages of ten biological replicates ± SE. Different upper-case letters indicate significant differences between MeJA treatments for the same soil conditions, according to Tukey’s test (*p* ≤ 0.01). Different lower-case letters indicate significant differences among soil conditions for the same MeJA treatment according to Tukey’s test (*p* ≤ 0.01).

	CO_2_ Assimilation (µmol CO_2_ m^−2^ s^−1^)	Stomatal Conductance (mol H_2_O m^−2^ s^−1^)	Transpiration (mmol H_2_O m^−2^ s^−1^)	SPAD (Chlorophyll Content Index)
Control	7.48 ± 0.66 Aa	0.65 ± 0.02 Aa	1.54 ± 0.05 Aa	22.9 ± 0.9 Aa
Control + MeJA	7.21 ± 0.35 Aa	0.65 ± 0.04 Aa	1.42 ± 0.05 Aa	22.3 ± 1.5 Ab
Al	7.10 ± 0.47 Aa	0.60 ± 0.04 Aa	1.42 ± 0.08 Aa	25.1 ± 2.3 Aa
Al + MeJA	8.25 ± 0.60 Aa	0.63 ± 0.05 Aa	1.55 ± 0.06 Aa	26.7 ± 2.1 Aa
Mn	7.25 ± 0.55 Aa	0.61 ± 0.04 Aa	1.58 ± 0.12 Aa	24.7 ± 0.9 Aa
Mn + MeJA	7.67 ± 0.30 Aa	0.65 ± 0.03 Aa	1.48 ± 0.11 Aa	22.6 ± 1.1 Ab
Al–Mn	7.06 ± 0.41 Aa	0.65 ± 0.00 Aa	1.53 ± 0.10 Aa	23.6 ± 1.6 Aa
Al–Mn + MeJA	7.45 ± 0.50 Aa	0.66 ± 0.02 Aa	1.59 ± 0.13 Aa	24.2 ± 0.9 Aab

**Table 2:** Aluminum and manganese concentrations and quality of fruits in highbush blueberry cv. Legacy plants subjected to different soil treatments (Al, Mn, and Al–Mn toxicity), and exogenous MeJA. All values represent averages of 10 biological replicates ± SE. Different upper-case letters indicate significant differences between MeJA treatments for the same soil conditions according to Tukey’s test (*p* ≤ 0.01). Different lower-case letters indicate significant differences among soil conditions for the same MeJA treatment according to Tukey’s test (*p* ≤ 0.01).

	Fruit Al Concentration (mg kg^−1^ DW)	Fruit Mn Concentration(mg kg^−1^ DW)	Equatorial Diameter (mm)	Soluble Solids (°Brix)	Firmness (gf mm^−1^)
Control	12.0 ± 1.0 Bd	4.7 ± 0.3 Bc	17 ± 0.2 Aa	14.3 ± 0.02 Aa	131 ± 6.6 Ab
Control + MeJA	20.8 ± 0.3 Ad	5.7 ± 0.3 Ac	15 ± 0.2 Ba	13.7 ± 0.2 Aab	137 ± 4.3 A
Al	32.5 ± 1.5 Bb	4.7 ± 0.8 Bc	16 ± 0.4 Ab	14.6 ± 0.5 Aa	141 ± 9.0 Aab
Al + MeJA	47.8 ± 3.4 Aa	6.8 ± 0.6 Ab	16 ± 0.2 Aa	13.2 ± 0.5 Bb	143 ± 3.3 A
Mn	22.5 ± 2.6 Bc	6.8 ± 0.6 Ab	16 ± 0.5 Ab	15.1 ± 0.1 Aa	133 ± 5.0 Bb
Mn + MeJA	27.2 ± 0.9 Ac	6.5 ± 0.5 Ab	16 ± 0.5 Aa	14.3 ± 0.5 Ba	157 ± 6.0 A
Al–Mn	47.8 ± 3.4 Aa	16.5 ± 0.9 Aa	16 ± 0.3 Ab	12.5 ± 0.1 Bb	150 ± 1.1 Ba
Al–Mn + MeJA	30.8 ± 0.8Bb	17.3 ± 0.3Aa	16 ± 0.8 Aa	14.2 ± 0.3 Aab	171 ± 5.8 A

## Data Availability

The data presented in this study are available in the results section.

## References

[1] Mora M.L., Schnettler B., Demanet R. (1999). Effect of liming and gypsum on soil chemistry, yield and mineral composition of ryegrass grown in an acidic Andisol. Commun. Soil Plant Anal..

[2] Bañados M.P., Ibáñez F., Toso A.M. (2009). Manganese toxicity induces abnormal shoot growth in ‘O’Neal’ blueberry. Acta Hortic..

[3] Reyes-Díaz M., Inostroza-Blancheteau C., Millaleo R., Cruces E., Wulff-Zottele C., Alberdi M., Mora M.L. (2010). Long-term aluminum exposure effects on physiological and biochemical features of highbush blueberry cultivars. J. Am. Soc. Hort. Sci..

[4] González-Villagra J., Escobar A., Ribera-Fonseca A., Carcamo M.P., Omena-Garcia R., Nunes-Nesi A., Inostroza-Blancheteau C., Alberdi M., Reyes-Díaz M. (2021). Differential mechanisms between traditionally established and new highbush blueberry (*Vaccinium corymbosum* L.) cultivars reveal new insights into manganese toxicity resistance. Plant Physiol. Biochem..

[5] Bojórquez-Quintal E., Escalante-Magaña C., Echeverría-Machado I., Martínez-Estévez M. (2017). Aluminum, a friend or foe of higher plants in acid soils. Front. Plant Sci..

[6] Kar D., Pradhan A., Datta S. (2020). The role of solute transporters in aluminum toxicity and tolerance. Physiol. Plant.

[7] Cárcamo-Fincheira P., Reyes-Díaz M., Omena-García R., Vargas J., Alvear M., Florez-Sarasa I., Rosado-Saouza L., Rengel Z., Fernie A., Nunes-Nesi A. (2021). Metabolomic analyses of highbush blueberry (*Vaccinium corymbosum* L.) cultivars revealed mechanisms of resistance to aluminum toxicity. Environ. Exp. Bot..

[8] Reyes-Díaz M., Alberdi M., Mora M.L. (2009). Short-term aluminum stress differentially affects the photochemical efficiency of photosystem II in highbush blueberry genotypes. J. Am. Soc. Hort. Sci..

[9] Inostroza-Blancheteau C., Rengel Z., Alberdi M., Mora M.L., Aquea F., Arce-Johnson P., Reyes-Díaz M. (2012). Molecular and physiological strategies to increase Al resistance in plants. Mol. Biol. Rep..

[10] Soto-Cerda B.J., Inostroza-Blancheteau C., Mathías M.M., Peñaloza E., Zuñiga J., Muñoz G., Rengel Z., Salvo-Garrido H. (2015). Marker-assisted breeding for TaALMT1, a major gene conferring aluminium tolerance to wheat. Biol. Plant.

[11] Kochian L.V., Hoekenga O.A., Pineros M.A. (2004). How do crop plants tolerate acid soils? Mechanisms of aluminum tolerance and phosphorous efficiency. Annu. Rev. Plant Biol..

[12] Millaleo R., Reyes-Díaz M., Ivanov A., Mora M.L., Alberdi M. (2010). Manganese as essential and toxic element for plants: Transport, accumulation and resistance mechanisms. J. Soil Sci. Plant Nutr..

[13] Alejandro S., Höller S., Meier B., Peiter E. (2020). Manganese in plants: From acquisition to subcellular allocation. Front. Plant Sci..

[14] Mora M.L., Rosas A., Ribera A.E., Rengel Z. (2009). Differential tolerance to Mn toxicity in perennial ryegrass genotypes: Involvement of antioxidative enzymes and root exudation of carboxylates. Plant Soil.

[15] Rojas Lillo Y., Alberdi M., Patricio A., Inostroza Blancheteau C., Rengel Z., Mora M.L., Reyes-Diaz M. (2014). Manganese toxicity and UV-B radiation differentially influence physiology and biochemistry of highbush blueberry cultivars. Funct. Plant Biol..

[16] Millaleo R., Alvear M., Aguilera P., González Villagra J., Mora M.L., Alberdi M., Reyes-Diaz M. (2020). Mn toxicity differentially affects physiological and biochemical features in highbush blueberry (*Vaccinium corymbosum* L.) cultivars. J. Soil Sci. Plant Nutr..

[17] Blair L.M., Taylor G.J. (1997). The nature of interaction between aluminum and manganese on growth and metal accumulation in *Triticum aestivum*. Environ. Exp. Bot..

[18] Yang Z.B., You J.F., Xu M.Y., Yang Z.M. (2009). Interaction between aluminum toxicity and manganese toxicity in soybean. Plant Soil.

[19] Nelson L.E. (1983). Tolerances of 20 rice cultivars to excess Al and Mn. Agron. J..

[20] Ahammed G.J., Xia X.J., Li X., Shi K., Yu J.Q., Zhou Y.H. (2015). Role of brassinosteroid in plant adaptation to abiotic stresses and its interplay with other hormones. Curr. Protein Pept. Sci..

[21] Wani S., Kumar V., Shriram V., Kumar Sah S. (2016). Phytohormones and their metabolic engineering for abiotic stress tolerance in crop plants. Crop J..

[22] Reyes-Díaz M., Lobos T., Cardemil L., Nunes-Nesi A., Retamales J., Jaakola L., Alberdi M., Ribera-Fonseca A. (2016). Methyl jasmonate: An alternative for improving the quality and health properties of fresh fruits. Molecules.

[23] Reyes-Díaz M., González-Villagra J., Figueroa C., Inostroza-Blancheteau C., Morales M., Bravo L.A., Ramakrishna A., Sirhindi G. (2021). Jasmonates and plant responses under metal stress. Jasmonates and Brassinosteroids in Plants: Metabolism, Signaling, Biotechnological Applications.

[24] Pérez A.G., Sanz C., Olías R., Olías J.M. (1997). Effect of methyl jasmonate on in vitro strawberry ripening. J. Agric. Food. Chem..

[25] Wang L., Stoner G. (2008). Anthocyanins and their role in cancer prevention. Cancer Lett..

[26] Sayyar M., Babalar M., Kalantari S., Martinez-Romero D., Guillén F., Serrano M., Valero D. (2011). Vapour treatments with methyl salicylate or methyl jasmonate alleviated chilling injury and enhanced antioxidant potential during postharvest storage of pomegranates. Food Chem..

[27] Yi T.G., Park Y., Park J.E., Park N. (2019). Enhancement of phenolic compounds and antioxidative activities by the combination of culture medium and methyl jasmonate elicitation in hairy root cultures of *Lactuca indica* L.. Nat. Product. Commun..

[28] Ulloa-Inostroza E.M., Alberdi M., Meriño-Gergichevich C., Reyes-Díaz M. (2017). Low doses of exogenous methyl jasmonate applied simultaneously with toxic aluminum improve the antioxidant performance of *Vaccinium corymbosum*. Plant Soil.

[29] Ribera-Fonseca A., Jiménez D., Leal P., Riquelme I., Roa J.C., Alberdi M., Peek R.M., Reyes-Díaz M. (2020). The anti-proliferative and anti-invasive effect of leaf extracts of blueberry plants treated with methyl jasmonate on human gastric cancer in vitro is related to their antioxidant properties. Antioxidants.

[30] Valle S.R., Carrasco J., Pinochet D., Calderini D.F. (2009). Grain yield, above-ground and root biomass of Al-tolerant and Al-sensitive wheat cultivars under different soil aluminum concentrations at field conditions. Plant Soil.

[31] Ulloa-Inostroza E.M., Alberdi M., Ivanov A.G., Reyes-Díaz M. (2019). Protective effect of methyl jasmonate on photosynthetic performance and its association with antioxidants in two blueberry cultivars with contrasting Al-resistance exposed to Al-toxic. J. Soil Sci. Plant Nutr..

[32] Hoffmann W.A., Poorter H. (2002). Avoiding bias in calculations of relative growth rate. Ann. Bot..

[33] Angélica S.R., María Adriana C.R., Rolando D.F., Hugo F.P., Renato G.Z., María de la Luz M.G., Alexander N. (2007). Métodos de Análisis de Tejidos Vegetales [en línea].

[34] Sarwar M., Kremer R.J. (1995). Determination of bacterially derived auxins using a microplate method. Lett. Appl. Microbiol..

[35] Glickmann E., Dessaux Y. (1995). A critical examination of the specificity of the salkowski reagent for indolic compounds produced by phytopathogenic bacteria. Appl. Environ. Microbiol..

[36] García-Plazaola J.I., Becerril J.M. (1999). A rapid HPLC method to measure lipophilic antioxidants in stressed plants: Simultaneous determination of carotenoids and tocopherols. Phytochem. Anal..

[37] Chinnici F., Bendini A., Gaiani A., Riponi C. (2004). Radical scavenging activities of peels and pulps from cv. Golden Delicious apples as related to their phenolic composition. J. Agric. Food Chem..

[38] Slinkard K., Singleton V.A. (1977). Total phenol analysis: Automation and comparison with manual methods. Am. J. Enol. Vitic..

[39] Du Z., Bramlage W.J. (1992). Modified thiobarbituric acid assay for measuring lipid peroxidation in sugar rich plant tissue extracts. J. Agric. Food Chem..

[40] Karla Y.P. (1998). Handbook of Reference Methods for Plant Analysis.

[41] Kochian L.V. (1995). Cellular mechanisms of aluminum toxicity and resistance in plants. Annu. Rev. Plant Physiol. Plant Mol. Biol..

[42] Lidon F., Barreiro M.G., Lauriano J. (1999). Effects of aluminium toxicity on nutrient accumulation in maize shoots: Implications on photosynthesis. J. Plant Nutr..

[43] Bose J., Babourina O., Ma Y., Zhou M., Shabala S., Rengel Z., Panda S.K., Baluska F. (2015). Specificity of ion uptake and homeostasis maintenance during acid and aluminium stresses in Aluminum Stress Adaptation in Plants. Signaling Communication in Plants.

[44] Reyes-Díaz M., Inostroza-Blancheteau C., Berríos G., Deppe M., Demanet R., Alberdi M. (2017). Physiological traits and Mn transporter genes expression in ryegrass genotypes under increasing Mn at short-term. Plant Physiol. Biochem..

[45] Wang W., Zhao X.Q., Hu Z.M., Shao J.F., Che J., Chen R.F., Dong X.Y., Shen R.F. (2015). Aluminium alleviates manganese toxicity to rice by decreasing root symplastic Mn uptake and reducing availability to shoots of Mn stored in roots. Ann. Bot..

[46] Muhammad N., Cai S., Shah J.M., Zhang G.P. (2016). The combined treatment of Mn and Al alleviates the toxicity of Al or Mn stress alone in barley. Acta Physiol. Plant.

[47] Cheong J.J., Do Choi Y. (2003). Methyl jasmonate as a vital substance in plants. Trends Genet..

[48] Chen X., Jiang W., Tong T., Chen G., Zeng F., Jang S., Gao W., Li Z., Mak M., Deng F. (2021). Molecular Interaction and Evolution of Jasmonate Signaling With Transport and Detoxification of Heavy Metals and Metalloids in Plants. Front. Plant Sci..

[49] Piotrowska-Niczyporuk A., Bajguz A., Kotowska U., Zambrzycka-Szelewa E., Sienkiewicz A. (2020). Auxins and cytokinins regulate phytohormone homeostasis and thiol-mediated detoxification in the green alga *Acutodesmus obliquus* exposed to lead stress. Sci. Rep..

[50] Anjum S.A., Wang A., Farooq M., Khan I., Xue L. (2011). Methyl jasmonate-induced alteration in lipid peroxidation, antioxidative defence system and yield in soybean under drought. J. Agron. Crop Sci..

[51] Mohamed H.I., Latif H.H. (2017). Improvement of drought tolerance of soybean plants by using methyl jasmonate. Physiol. Mol. Biol. Plants.

[52] Mendoza D., Cuaspud O., Arias J.P., Ruiz O., Arias M. (2018). Effect of salicylic acid and methyl jasmonate in the production of phenolic compounds in plant cell suspension cultures of *Thevetia peruviana*. Biotechnol. Rep..

[53] Maciejewska B., Kopcewicz J. (2002). Inhibitory effect of methyl jasmonate on flowering and elongation growth in *Pharbitis nil*. J. Plant Growth Regul..

[54] Farooq M.A., Gill R.A., Islam F., Ali B., Liu H., Xu J., He S., Zhou W. (2016). Methyl jasmonate regulates antioxidant defense and suppresses arsenic uptake in *Brassica napus* L.. Front. Plant Sci..

[55] Per T.S., Khan N.A., Masood A., Fatma M. (2016). Methyl jasmonate alleviates cadmium-induced photosynthetic damages through increased S-assimilation and glutathione production in mustard. Front. Plant Sci..

[56] Li T., Jiang Z., Zhang L., Tan D., Wei Y., Yuan H., Li T., Wang A. (2016). Apple (*Malus domestica*) MdERF2 negatively affects ethylene biosynthesis during fruit ripening by suppressing MdACS1 transcription. Plant J..

[57] Flores G., Ruiz del Castillo M.L. (2014). Influence of preharvest and postharvest methyl jasmonate treatments on flavonoid content and metabolomic enzymes in red raspberry. Postharvest Biol. Technol..

[58] Kucuker E., Ozturk B., Celik S.M., Aksit H. (2014). Pre-harvest spray application of methyl jasmonate plays an important role in fruit ripening, fruit quality and bioactive compounds of *Japanese plums*. Sci. Hortic..

[59] Martínez-Esplá A., Zapata P.J., Castillo S., Guillén F., Martínez-Romero D., Valero D., Serrano M. (2014). Preharvest application of methyl jasmonate (MeJA) in two plum cultivars. 1. Improvement of fruit growth and quality attributes at harvest. Postharvest Biol. Technol..

[60] Zapata P.J., Martínez-Esplá A., Guillén F., Díaz-Mula H.M., Martínez-Romero D., Serrano M., Valero D. (2014). Preharvest application of methyl jasmonate (MeJA) in two plum cultivars. 2. Improvement of fruit quality and antioxidant systems during postharvest storage. Postharvest Biol. Technol..

[61] Hua Z., Zhen-Yu W., Xin Y., Hai-Tian Z., Ying-Chun Z., Ai-Jun D., Jing J., Jing W. (2014). Determination of free amino acids and 18 elements in freeze-dried strawberry and blueberry fruit using an Amino Acid Analyzer and ICP-MS with micro-wave digestion. Food Chem..

[62] Zeiner M., Cindrić I.J. (2018). Harmful elements (Al, Cd, Cr, Ni, and Pb) in wild berries and fruits collected in Croatia. Toxics.

[63] Masahiro K., Kato-Negish M. (2011). Link between aluminum and the pathogenesis of Alzheimer’s disease: The integration of the aluminum and amyloid cascade hypotheses. Int. J. Alzheimers Dis..

[64] FAO/WHO (2011). Evaluation of Certain Food Additives and Contaminants: Seventy-Fourth Report of the Joint FAO/WHO Expert Committee on Food Additives.

[65] Fernández-Maestre R. (2014). Aluminum: Intake, absorption, excretion and toxicity. Rev. Costarr. Publica.

